# Supplementary material for: A Health Guidance App to Improve Motivation, Adherence to Lifestyle Changes and Indicators of Metabolic Disturbances among Japanese Civil Servants
Source: Int J Environ Res Public Health. 2020 Nov 4;17(21):8147. doi: 10.3390/ijerph17218147 (PMC7662815; doi:10.3390/ijerph17218147)
Supplement: Supplementary file 1 [file ijerph-17-08147-s001.pdf]

Supplemental Table S1. Diagnostic criteria for metabolic syndrome in Japan.

No.	Risk factor	Cut-off points
1	Waist circumference	
	Male	≥ 85 cm
	Female	≥ 90 cm
2	Triglyceride	≥ 150 mg/dl
	or	
	HDL-cholesterol	< 40 mg/dl
3	Blood pressure	
	Systolic BP	≥ 130 mmHg
	or/and	
	Diastolic BP	≥ 85 mmHg
4	Fasting Blood Glucose	≥ 110㎎/dl

Note: The Metabolic Syndrome Diagnostic Criteria Review Committee of Japan defines metabolic syndrome as 1 is required, having two or more of 2 to 4; HDL: High-density lipoprotein; BP: Blood pressure.

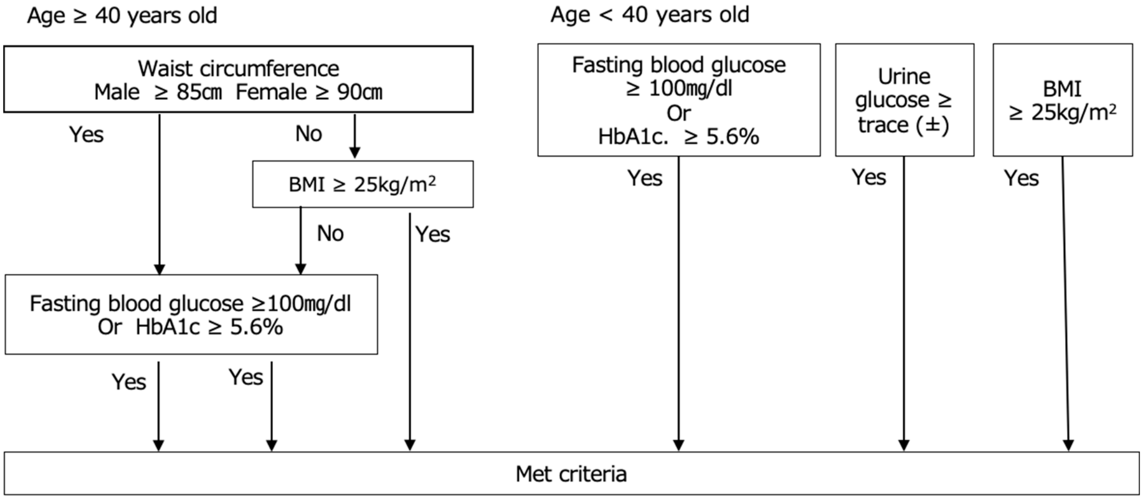

Supplemental Figure S1. Inclusion criteria for participants.

Note: BMI: Body mass index; HbA1c: Glycated hemoglobin.
